# Liver-Derived IGF-I Regulates Mean Life Span in Mice

**DOI:** 10.1371/journal.pone.0022640

**Published:** 2011-07-25

**Authors:** Johan Svensson, Klara Sjögren, Jenny Fäldt, Niklas Andersson, Olle Isaksson, John-Olov Jansson, Claes Ohlsson

**Affiliations:** 1 Department of Internal Medicine, Sahlgrenska University Hospital, Göteborg, Sweden; 2 Institute of Neuroscience and Physiology, Sahlgrenska Academy, University of Gothenburg, Göteborg, Sweden; 3 Food Science Unit, Department of Chemical and Biological Engineering, Chalmers University of Technology, Göteborg, Sweden; City of Hope National Medical Center and Beckman Research Institute, United States of America

## Abstract

**Background:**

Transgenic mice with low levels of global insulin-like growth factor-I (IGF-I) throughout their life span, including pre- and postnatal development, have increased longevity. This study investigated whether specific deficiency of liver-derived, endocrine IGF-I is of importance for life span.

**Methods and Findings:**

Serum IGF-I was reduced by approximately 80% in mice with adult, liver-specific IGF-I inactivation (LI-IGF-I^-/-^ mice), and body weight decreased due to reduced body fat. The mean life span of LI-IGF-I^-/-^ mice (n = 84) increased 10% vs. control mice (n = 137) (Cox's test, p<0.01), mainly due to increased life span (16%) of female mice [LI-IGF-I^-/-^ mice (n = 31): 26.7±1.1 vs. control (n = 67): 23.0±0.7 months, p<0.001]. Male LI-IGF-I^-/-^ mice showed only a tendency for increased longevity (p = 0.10). Energy expenditure, measured as oxygen consumption during and after submaximal exercise, was increased in the LI-IGF-I^-/-^ mice. Moreover, microarray and RT-PCR analyses showed consistent regulation of three genes (heat shock protein 1A and 1B and connective tissue growth factor) in several body organs in the LI-IGF-I^-/-^ mice.

**Conclusions:**

Adult inactivation of liver-derived, endocrine IGF-I resulted in moderately increased mean life span. Body weight and body fat decreased in LI-IGF-I^-/-^ mice, possibly due to increased energy expenditure during exercise. Genes earlier reported to modulate stress response and collagen aging showed consistent regulation, providing mechanisms that could underlie the increased mean life span in the LI-IGF-I^-/-^ mice.

## Introduction

Insulin-like growth factor-I (IGF-I) is among the relatively few factors that regulate life span. *Caenorhabditis elegans* (*C. elegans*, a nematode) and *Drosophila melanogaster* (fruit fly) have common signaling pathways for IGF-I and insulin, and disruption of IGF-I/insulin signaling in these species increases longevity [1]–[4]. Mice have separate receptors for IGF-I and insulin, and longevity increases in several mouse strains with low global activity of the growth hormone (GH)/IGF-I axis. Spontaneous mutations in Prop-1 (Ames dwarf mice) [5] and Pit-1 (Snell dwarf mice) [6] result in impaired pituitary gland development, low GH secretion, and increased life expectancy [5], [6]. Life expectancy increases in mice with deficient GH-releasing hormone receptor (*Ghrhr* ^lit/lit^ mice) [6], GH receptor null mice [7], and mice that are heterozygous in terms of inactivation of the IGF-I receptor in total body (*Igf1r* ^+/−^ mice) [8]. Longevity also increases in mouse models with reduced IGF-I bioavailability due to knockout of the IGF binding protein (IGFBP) specific protease PAPP-A [9], and in mice with defective IGF-I signaling downstream of the IGF-I receptor such as mice lacking the IGF-I receptor substrate p66^shc^ [10] and mice that overexpress KLOTHO [11]. A cross-sectional study of 31 strains of mice detected an inverse correlation between plasma IGF-I levels at 6 months and median lifespan [12]. In terms of the brain, the role of IGF-I is complex and the effects of IGF-I on lifespan and neuronal function can be uncoupled to some extent [13]. In mice, administration of IGF-I may provide protection from neurodegeneration [14] while heterozygous brain-specific IGF-I receptor knockout enhances longevity [15].

The rate of aging is plastic and mutations in genes influencing endocrine signaling, fertility, stress responses, metabolism and telomeres can affect life span in model organisms [16], [17]. Reduced body weight may participate importantly in the increased life span of long-lived, GH-deficient Ames dwarf mice, as could low fertility [18]. However, several studies have demonstrated that reduced body size or fertility is not required for increased life span in mice with low GH/IGF-I activity [8], [18]. Reduced activity of nutrient-sensing pathways, such as attenuated IGF-I signaling, may mediate some of the anti-aging effects of dietary restriction in mice [19]. However, the prolonging effect on life span by low food intake is partly independent of IGF-I as caloric restriction prolongs life span even further in Ames dwarf mice [20]. Furthermore, caloric restriction reduces the mitochondrial production of reactive oxidative species (ROS) and oxidative damage [21]. In *C. elegans* and *Drosophila melanogaster* with low IGF-I/insulin activity [17], [22], [23], as well as in *Igf1r* ^+/−^ mice [8] and mice lacking the IGF-I receptor substrate p66^shc^ [10], increased resistance to oxidative stress might be a mechanism underlying the longer life span.

The major part of serum IGF-I is liver-derived [24]–[26]. In addition to regulation by GH [26], serum IGF-I levels are also affected by food intake, exercise and age [26]. A mouse model with liver-specific, inducible inactivation of the IGF-I gene, using the Cre-LoxP conditional knockout system, has been developed (LI-IGF-I^-/-^ mice) [24], [26]–[30]. The selective inactivation of the IGF-I gene in the liver results in a 75-80% reduction in serum IGF-I, whereas the expression of IGF-I is unaffected in other tissues [24]. GH secretion is increased in LI-IGF-I^-/-^ mice secondary to a decreased negative feedback by serum IGF-I [24], [29].

Although LI-IGF-I^-/-^ mice have no major disturbance in postnatal longitudinal bone growth [24], cortical bone mass is clearly decreased [26], [28], [31]. Increased peripheral resistance in LI-IGF-I^-/-^ mice results in elevated blood pressure [30]. Blood glucose is normal but circulating levels of insulin and cholesterol are increased [27]. The present study assessed the role of adult expression of liver-derived IGF-I in life span, and also investigated the importance of deficiency of liver-derived IGF-I for parameters associated with life span including fertility, body composition, oxygen consumption, activity level, food intake, and expression of longevity-associated genes in several organs.

## Results

Liver-specific inactivation of the IGF-I gene was induced in mice at one month of age in most experiments. However, food intake, body composition, oxygen consumption at rest, and activity level were measured in mice that underwent inactivation of liver-derived IGF-I at 12 months of age. In the experiments determining life span, serum IGF-I was 321±5 ng/ml in the control mice (n = 137) and 62±3 ng/ml in the LI-IGF-I^-/-^ mice (n = 84), an 81% reduction (p<0.001). In all other experiments, serum IGF-I concentration decreased approximately 80% in the various groups of LI-IGF-I^-/-^ mice (p<0.001 in all experiments; data not shown).

### Body weight and life span

**Figure 1:**
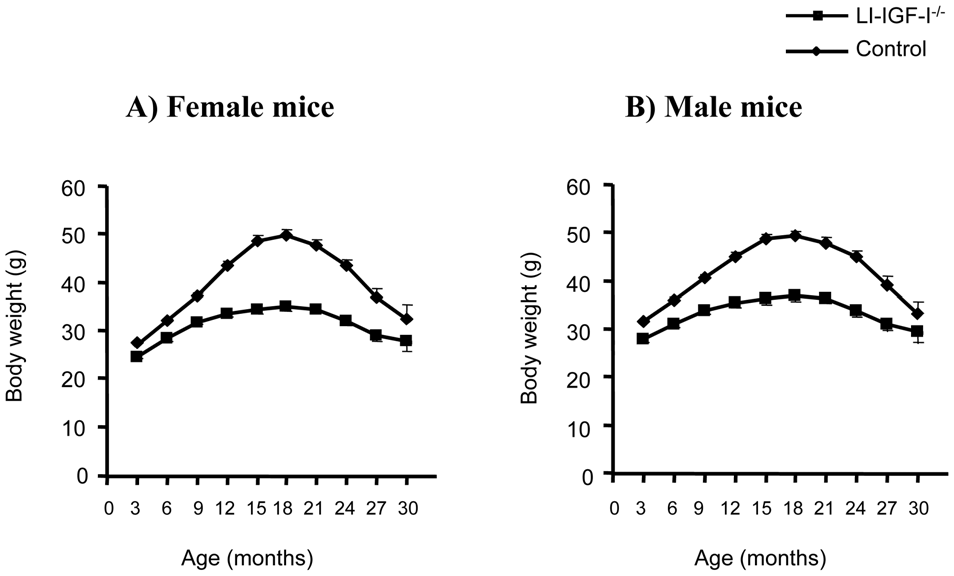
Reduced body weight in LI-IGF-I^-/-^ mice. Body weight in A) female and B) male mice. Body weight was determined every third month during 3-30 months of age. Between 3 and 27 months of age, body weight was significantly lower in LI-IGF-I^-/-^ compared with control mice in the total number of mice and also in the male and female subgroups (all analyses p<0.001). The vertical bars indicate the SE for the mean values shown.

Body weight was measured every third month between 3 and 30 months of age. Reduced body weight was observed in both female (Fig. 1A) and male (Fig. 1B) LI-IGF-I^-/-^ mice.

**Figure 2:**
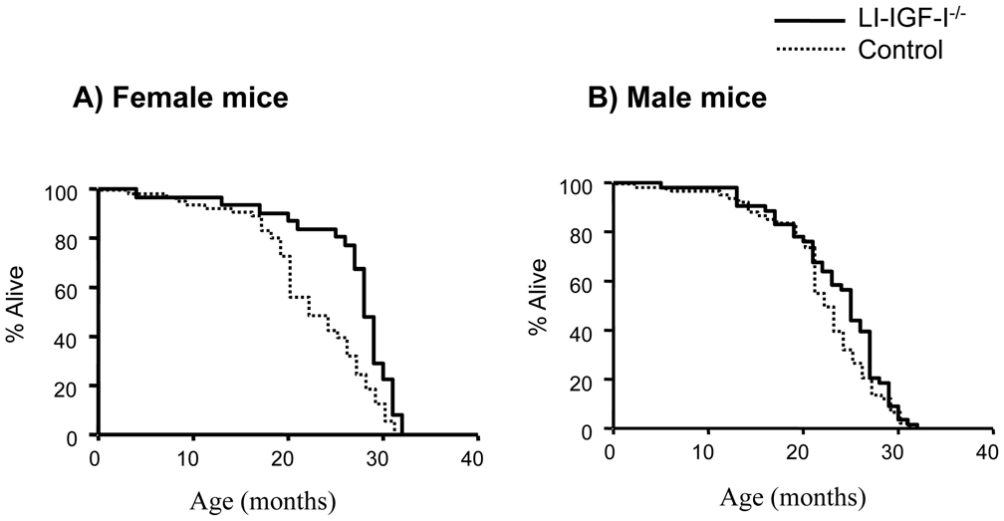
Increased mean life span of female LI-IGF-I^-/-^ mice. A) Female LI-IGF-I^-/-^ mice live 16% longer than female control mice (26.7 ± 1.1 vs. 23.0 ± 0.7 months, p<0.01 using unpaired *t*-test; Kaplan-Meyer analysis p<0.001, Cox's test). B) Although not statistically significant, male LI-IGF-I^-/-^ mice live 6% longer than male control mice (23.5 ± 0.8 vs. 22.1 ± 0.7 months, p = 0.17 using unpaired t-test; Kaplan-Meyer analysis: p = 0.10, Cox's test).

Mean life span increased 10% [LI-IGF-I^-/-^ mice (n = 84): 24.7±0.6 months vs. control (n = 137): 22.5±0.5 months, p<0.01 using unpaired *t*-test; Kaplan-Meier analysis: p = 0.001, Cox's test], mainly due to increased life span (16%) in female mice [LI-IGF-I^-/-^ mice (n = 31): 26.7±1.1 vs. control (n = 67): 23.0±0.7 months, p<0.01 using unpaired *t*-test; Kaplan-Meyer analysis p<0.001, Cox's test; Fig. 2A]. Male mice showed only a tendency toward increased mean life span [6% increase; LI-IGF-I^-/-^ mice (n = 53): 23.5±0.8 vs. control (n = 70): 22.1±0.7 months, p = 0.17 using unpaired *t*-test; Kaplan-Meyer analysis p = 0.10, Cox's test; Fig 2B].

Maximal life span of LI-IGF-I^-/-^ mice was not significantly different compared to that of control mice (p = 0.11). In male LI-IGF-I^-/-^ mice, maximal life span was similar as that in control mice (p = 0.92) whereas in female LI-IGF-I^-/-^ mice, there was a non-significant tendency to increased maximum life span. When 90% of the joint distribution of female mice had died, 19% (6/31) of female LI-IGF-I^-/-^ mice and 6% (4/67) of female control mice were still alive (p = 0.07).

### Fertility

**Figure 3:**
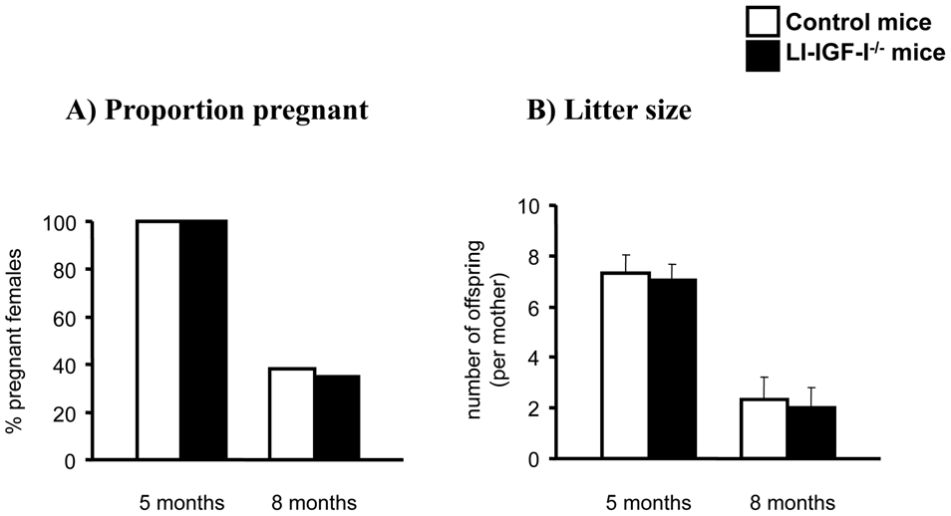
Normal fertility in female LI-IGF-I^-/-^ mice. A) The percentage of pregnant female mice in relation to the total number of female mice in each group. B) Litter size when female LI-IGF-I^-/-^ mice were mated with age-matched male control mice at 5 and 8 months of age (n = 12-14 in each group). The experiments showed normal fertility in female LI-IGF-I^-/-^ mice. In Figure 3B, the error bars indicate the standard error for the mean values shown.

When 5- and 8-month-old female control and LI-IGF-I^-/-^ mice were mated with male controls of similar age, the proportion of pregnant females and the litter size were similar in both groups (Fig. 3A and B). Similarly, when 5- and 8-month-old male control and LI-IGF-I^-/-^ mice were mated with female controls of similar age, the proportion of pregnant females and the litter size were similar in both groups (data not shown).

### Body composition

We previously demonstrated reduced body weight due to reduced body fat in 13-month-old male and female LI-IGF-I^-/-^ mice that underwent inactivation of liver-derived IGF-I at one month of age [27]. The present study extends those findings by investigating the effect of inactivation of IGF-I in hepatocytes at 12 months of age. Six months later, at 18 months of age, measurements using dual-energy X-ray absorptiometry (DEXA) showed reduced body fat also after correction for the reduced body weight in the LI-IGF-I^-/-^ mice as compared with the control mice (Table 1). Absolute values of lean mass was unchanged whereas after correction for body weight, lean mass even increased in the LI-IGF-I^-/-^ mice (Table 1). Dissections of fat pads showed lower weight of inguinal, retroperitoneal, and gonadal fat in the LI-IGF-I^-/-^ mice as compared with the control mice (Table 1).

**Table 1:** Liver-derived IGF-I were inactivated at 12 months of age.

	Control mice	LI-IGF-I^-/-^ mice	P-value
Body weight (g)	39.9 (0.9)	30.0 (2.5)	0.01
**DEXA**			
Body fat (g)	10.9 (1.9)	5.4 (1.2)	<0.05
Body fat (%)	29.3 (4.0)	18.9 (2.3)	<0.05
Lean mass (g)	24.4 (1.4)	21.3 (1.8)	0.22
Lean mass (%)	70.7 (4.0)	81.1 (2.3)	<0.05
**Absolute weight of fat pads**			
Inguinal fat (g)	0.69 (0.07)	0.27 (0.10)	0.01
Retroperitoneal fat (g)	0.25 (0.05)	0.02 (0.01)	<0.01
Gonadal fat (g)	0.40 (0.15)	0.05 (0.03)	<0.05
**Relative weight of fat pads**			
Inguinal fat (% of BW)	1.72 (0.16)	0.80 (0.25)	<0.05
Retroperitoneal fat (% of BW)	0.61 (0.13)	0.07 (0.02)	<0.01
Gonadal fat (% of BW)	0.99 (0.35)	0.15 (0.07)	<0.05

In control (n = 6) and LI-IGF-I^-/-^ (n = 8) mice, at 18 months of age, measurements using DEXA and dissections of fat pads were performed. The DEXA scans were from the lower level of the head to the inferior level of the first tail vertebra (the maximal scan area of the DEXA). Values are given as means (SEM).

BW  =  body weight.

### Food intake, activity level, and oxygen consumption

We observed no differences in food intake, feces output, oxygen consumption at rest, and spontaneous activity level while measuring 13-month-old female mice when body weight was still similar in both groups (Table 2). However, at 18 months, body weight was reduced in the female LI-IGF-I^-/-^ mice compared with the female control mice (p<0.001; data not shown). Additional experiments to determine oxygen consumption during rest in 24-month-old female control (n = 8) and LI-IGF-I^-/-^ (n = 5) mice that underwent inactivation of liver-derived IGF-I at one month of age showed no between-group difference (data not shown).

**Table 2:** Liver-derived IGF-I were inactivated at 12 months of age.

	Control mice	LI-IGF-I^-/-^ mice	P-value
**13-month-old female mice**			
Body weight (g)	38.8 (1.9)	38.6 (1.6)	0.94
Food intake	3.23 (0.28)	3.42 (0.24)	0.60
Feces output	1.26 (0.12)	1.25 (0.11)	0.95
Activity level (counts/min)	3.57 (0.51)	3.78 (0.55)	0.78
VO_2_ (ml/min/kg ^0.75^)	13.8 (0.9)	14.1 (0.5)	0.76
RER (VCO_2_/VO_2_)	0.70 (0.01)	0.71 (0.01)	0.17

At 13 months of age, before there was any difference in body weight, food intake, feces output, activity level, and oxygen consumption at rest were determined in female control (n = 8) and LI-IGF-I^-/-^ (n = 8) mice. Values are given as means (SEM).

**Figure 4:**
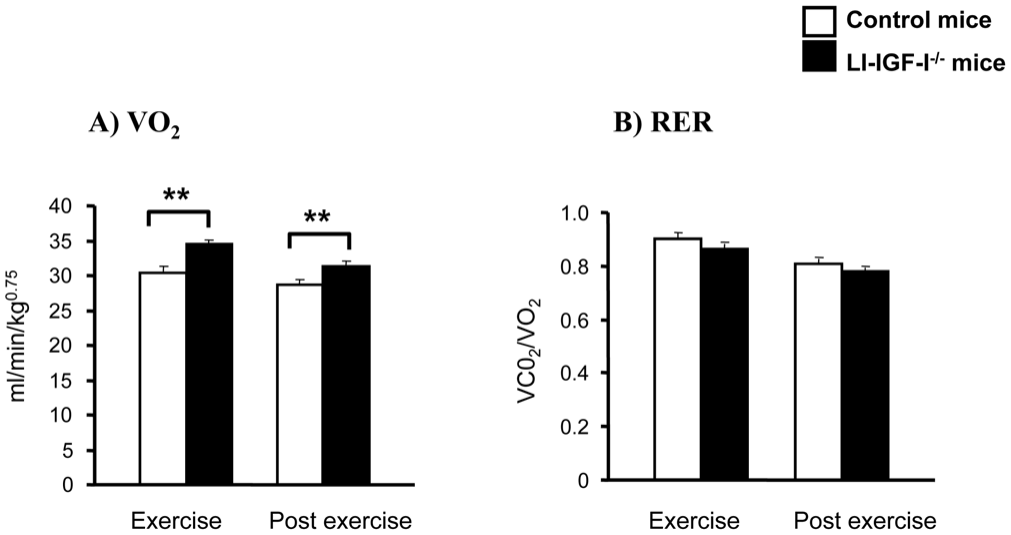
Increased oxygen consumption during submaximal exercise and post-exercise in LI-IGF-I^-/-^ mice. A) The volume of O_2_ consumed (VO_2_) and B) respiratory exchange ratio (RER) during submaximal exercise (30 min) and post-exercise (20 min) in 18-month-old female control (n = 7) and LI-IGF-I^-/-^ (n = 7) mice. The vertical bars indicate the SE for the mean values shown. ** p<0.01 vs. control mice

To further investigate mechanisms underlying reduced body fat in LI-IGF-I^-/-^ mice, oxygen consumption during submaximal exercise and post exercise was determined in 18-month-old female control and LI-IGF-I^-/-^ mice that underwent inactivation of liver-derived IGF-I at one month of age. The measurements showed that oxygen consumption was increased during submaximal exercise and post-exercise in LI-IGF-I^-/-^ mice compared with controls (Fig. 4A). Respiratory exchange ratio (RER) was unchanged (Fig. 4B).

### Microarray analysis

We performed DNA microarray analyses in female control and LI-IGF-I^-/-^ mice to identify possible mechanisms underlying increased life span. We sought to determine whether there were alterations in the global gene expression pattern that occurred consistently in various tissues, including liver, skeletal muscle, heart, fat (retroperitoneal fat) and bone (vertebrae). Four genes were regulated according to the criteria described in Materials and Methods (Table 3). Heat shock protein 1A (Hspa1a), heat shock protein 1B (Hspa1b), and connective tissue growth factor (Ctgf) were decreased in all organs that expressed these genes (Table 3). CCAAT/enhancer protein delta (Cebpd) decreased in 4 of the 5 analyzed organs (Table 3). The expression of the IGF-I receptor was unchanged in all organs studied (data not shown).

**Table 3:** Genes regulated in liver, skeletal muscle, heart, fat (retroperitoneal fat), and bone (vertebrae) in 7-month-old female control (n = 6) and LI-IGF-I^-/-^ (n = 7) mice as determined by DNA microarray analyses.

			Liver		Muscle		Heart		Fat		Bone	
Gene title	Gene symbol	GeneBank Accession	FC	Exp	FC	Exp	FC	Exp	FC	Exp	FC	Exp
Heat shock protein 1A	Hspa1a	M12571	ND	ND	-2.48	**	-2.69	**	-2.30	**	-1.70	**
Heat shock protein 1B	Hspa1b	AF109906	-2.73	**	-2.53	**	ND	ND	-2.08	***	-1.51	**
Connective tissue growth factor	Ctgf	M70642	ND	ND	-2.12	**	-1.53	***	-2.79	**	-1.78	***
CCAAT/enhancer protein delta (C/EBP delta)	Cebpd	X61800	-2.41	*	-2.06	***	NC	**	-1.87	***	-1.57	**

The regulation of mRNA levels of different genes is indicated by the average-fold change (FC) according to the rules given in Materials and Methods.

Exp  =  Expression; *, 0–500; ** 501–2000; *** 2001–8000 (Arbitrary units by Affymetrix).

NC indicates no change (according to the rules given in Materials and Methods) and ND indicates not detectable.

### RT-PCR

**Figure 5:**
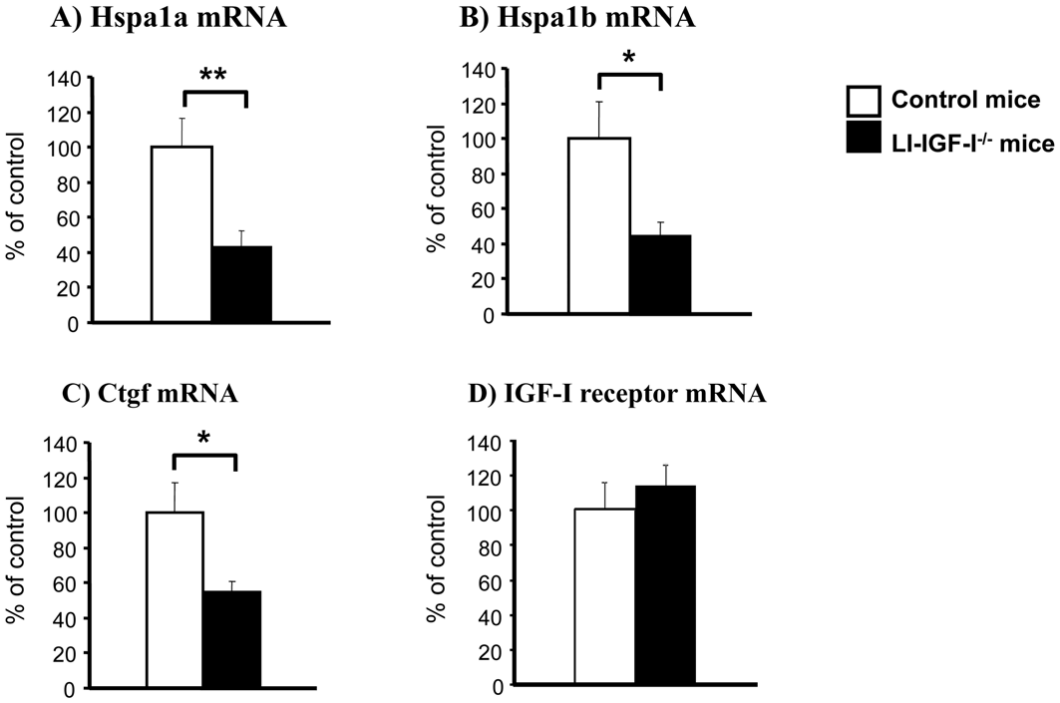
Decreased mRNA levels of Hspa1a, Hspa1b, and Ctgf in LI-IGF-I^-/-^ mice. To confirm the microarray findings, RT-PCR analyses were performed on skeletal muscle (triceps) samples from individual mice. The RT-PCR analyses showed reduced mRNA levels of A) heat shock protein 1A (Hspa1a), B) heat shock protein 1B (Hspa1b), and C) connective tissue growth factor (Ctgf). D) IGF-I receptor mRNA level was unchanged. The mRNA levels in LI-IGF-I^-/-^ mice (n = 7) are expressed as percent of that in control mice (n = 6). The vertical bars indicate the SE for the mean values shown.* p<0.05 ** p<0.01 vs. control mice.

To confirm the microarray findings, we performed RT-PCR analyses on skeletal muscle (triceps) samples from individual mice. The RT-PCR analyses confirmed that mRNA levels of Hspa1a (Fig. 5A), Hspa1b (Fig. 5B), and Ctgf (Fig. 5C) decreased in LI-IGF-I^-/-^ mice while mRNA levels of Cebpd were unchanged (data not shown). The mRNA level of the IGF-I receptor was similar in LI-IGF-I^-/-^ and control mice (Fig. 5D).

## Discussion

This study shows that deficiency of liver-derived, endocrine IGF-I results in increased mean life span. Mean life span increased significantly in female LI-IGF-I^-/-^ mice and both genders combined, while male LI-IGF-I^-/-^ mice showed only a tendency for increased survival. Body weight decreased in LI-IGF-I^-/-^ mice due to reduced fat mass. We observed no between-group differences in fertility, food intake, and activity level, whereas oxygen consumption during and after submaximal exercise was increased in LI-IGF-I^-/-^ mice. Gene expression analyses of several tissues in LI-IGF-I^-/-^ mice identified consistent regulation of three transcripts (Hspa1a, Hspa1b, and Ctgf) that might be involved in the aging process.

Low activity of IGF-I in nematodes [1], [2], fruit flies [3], [4], and mice [5]–[8], [15] results in increased life span. However, the role of IGF-I in adult life is difficult to evaluate in these animals due to the possible effect of absence of IGF-I activity during pre- and postnatal development. Furthermore, these previous studies did not evaluate the relative importance of liver-derived circulating IGF-I vs. that of IGF-I produced in peripheral tissues. In LI-IGF-I^-/-^ mice of this study, the deficiency of endocrine, liver-derived IGF-I was induced at 1 or 12 months of age. Consequently, the mice likely developed normally but then underwent a maintained, selective inactivation of IGF-I in hepatocytes, but retained normal IGF-I expression in the peripheral tissues as shown in several previous studies [24], [27]–[30], [32]–[35]. Therefore, the present study is the first to investigate life span in mice with normal IGF-I levels during development.

The extension of mean life span in LI-IGF-I mice with inactivation of liver-derived IGF-I was moderate (+10%), less than that observed in GH-deficient Ames and Snell dwarf mice [5], [6]. However, in contrast to the long-lived dwarf mice, longitudinal growth is almost normal in LI-IGF-I mice and body weight decreases only moderately [24], [27]. The results of some studies suggest that the availability of food [36] and GH status [37] during the first days or weeks of life can affect longevity in mice. Therefore, unaffected IGF-I levels during the first month of life before inactivation of liver-derived IGF-I could be of importance for the less marked extension of mean life span in LI-IGF-I^-/-^ mice compared to Ames and Snell dwarf mice that have low GH and IGF-I levels also during early postnatal development [5], [6]. Furthermore, the LI-IGF-I^-/-^ mice display compensatory increased GH levels [geometric mean plasma GH concentration 3.1-fold higher than that in control mice [24], [26], [29]]. This increased GH secretion may exert a negative effect on life span in LI-IGF-I mice and the full importance of liver-derived circulating IGF-I for life span regulation is therefore difficult to evaluate. However, our study shows that a marked reduction of liver-derived circulating IGF-I, combined with a moderate increase in circulating GH levels, results in a small but significant increase in mean life span.

Mean life span increased significantly (16%) in female LI-IGF-I^-/-^ mice, while male LI-IGF-I^-/-^ mice showed only a non-significant trend toward increased longevity. Similarly, life span in *Igf1r* ^+/-^ mice increased significantly only in female mice [8]. A human study showed decreased body height and reduced activity of the IGF-I receptor in female but not male offspring of long-lived centenarians [38]. Furthermore, the consequences of low circulating GH/IGF-I values differ considerably between men and women with hypopituitary disease [39]. Therefore, the importance of IGF-I for life span regulation appears to be partly gender-dependent across species.

Reduced body weight in LI-IGF-I^-/-^ mice could be one mechanism for prolonged life span in these mice. Previous studies demonstrated gradually reduced body fat in LI-IGF-I^-/-^ mice, at least partly mediated by the compensatory increase in GH secretion [27]. In a previous study, body fat was unchanged after correction for the reduced body weight in mice with deficiency of liver-derived IGF-I [40]. In the present study, late inactivation of liver-derived endocrine IGF-I (at 12 months of age) resulted in reduced body weight and decreased body fat also after correction for body weight. This late inactivation of IGF-I in hepatocytes may resemble the age-related decline in serum IGF-I observed in elderly humans more closely than early inactivation of liver-derived IGF-I in previous mouse models.

We detected no between-group differences in food intake, oxygen consumption at rest, or activity level, whereas oxygen consumption during submaximal exercise was increased in LI-IGF-I^-/-^ mice. We previously observed an association between reduced energy expenditure during submaximal exercise and subsequent body fat accumulation in mice with global deficiency of interleukin-6 [41], [42]. Furthermore, LI-IGF-I^-/-^ mice bear some resemblance to fat-specific insulin receptor knockout (FIRKO) mice, which display leanness, increased energy expenditure, and enhanced longevity [43], [44].

Since fertility of both male and female mice was similar to the controls both in this study and a previous study of mice with deficiency of liver-derived IGF-I [25], changes in fertility did not cause increased mean life span in LI-IGF-I^-/-^ mice. Furthermore, changes in cardiovascular risk factors likely do not explain increased longevity in the LI-IGF-I^-/-^ mice, because these mice have increased blood pressure [30] and increased circulating levels of insulin and cholesterol [27]. Insulin resistance and hyperinsulinemia are associated with increased ROS formation, a pro-inflammatory milieu, and a reduction of life span [23]. Impaired phosphoinositide 3-kinase pathway, decreased NAD-dependent deacetylase sirtuin (Sirt) 1 activity, and telomere shortening may mediate the reduction of life span in conditions of hyperinsulinemia/insulin resistance [23]. Furthermore, increased circulating levels of insulin may activate the IGF-I receptor [23]. In the present study, the mRNA level of the IGF-I receptor was unchanged in LI-IGF-I^-/-^ mice in all organs studied using microarray and in skeletal muscle as measured by RT-PCR. In another mouse model of deficiency of liver-derived IGF-I with approximately similar phenotype as that of LI-IGF-I^-/-^ mice including insulin resistance [25], [26], IGF-I receptor mRNA levels were similar as those in control mice in multiple tissues [45]. Tyrosine phosphorylation state of the IGF-I receptor in skeletal muscle was approximately similar in mice with deficiency of liver-derived IGF-I and control mice before and after IGF-I administration in one study [46], but otherwise little is known of the extent to which the IGF-I receptor is activated in mice with deficiency of liver-derived IGF-I. Therefore, the possibility cannot be excluded that increased insulin levels in LI-IGF-I^-/-^ mice, by an activation of the IGF-I receptor or by other effects, could contribute to the less pronounced extension of life span in these mice compared to Ames and Snell dwarf mice or *Igf1r* ^+/−^ mice.

Microarray analyses performed to identify genes regulated by liver-derived IGF-I studied only female mice because mean life span increased significantly in female but not male LI-IGF-I^-/-^ mice. Since we sought transcripts that were consistently regulated in several tissues, we analyzed tissue samples from liver, skeletal muscle, heart, fat (retroperitoneal fat) and bone (vertebrae). Three consistently regulated genes (Hspa1a, Hspa1b, and Ctgf) were identified and confirmed by RT-PCR analyses.

The Hspa1a and Hspa1b genes of LI-IGF-I^-/-^ mice were downregulated in all tissues that expressed these genes. Hspa1a and Hspa1b encode members of the heat shock protein 70 (Hsp70) family. Hsp70 proteins are stressed-induced chaperones involved in cellular repair and maintenance mechanisms [47]. In *Drosophila melanogaster*, experimentally induced overexpression of Hsp70 prolongs life span [48]. However, expression of endogenous Hsp70 after heat stress exposure is reduced in long-lived *Drosophila melanogaster* resistant to heat shock [49]. In humans, increased Hsp70 expression has been implicated in the development of atherosclerosis [50] and may also participate in the progression of some cancers [51]. Therefore, drugs that modulate the heat shock response are being developed [51]. In long-lived dwarf mice, the regulation of heat shock proteins is complex and partly organ-specific, but in accord with the present results, some *in vitro* and *in vivo* data suggest reduced expression of heat shock proteins in dwarf mice with low GH/IGF-I activity [52], [53]. Therefore, inhibition of genes encoding heat shock proteins may be of importance for the increased life span of mice with low IGF-I activity, which is in some accordance with the hypothesis that longevity correlates with lower basal levels of heat shock protein gene expression and more robust heat shock response [54]. An effect of the GH/IGF-I axis on stress systems also somewhat concurs with previous findings that nematodes and fruit flies [17], [22], [23] as well as mouse strains with low IGF-I activity [8], [10], including mice with deficiency of liver-derived IGF-I [55], display increased resistance to oxidative stress.

The mRNA levels of Ctgf were also affected in the LI-IGF-I^-/-^ mice. Abnormal amplification of Ctgf-dependent signals results in failure to terminate tissue repair, leading to pathological scarring in conditions such as fibrosis and cancer [56]. It is well known that GH treatment increases serum IGF-I level as well as collagen formation [57]. Long-lived Snell dwarf mice with low GH/IGF-I activity have delayed collagen aging in terms of age-dependent collagen cross-linking [6]. Thus, inhibition of Ctgf expression by deficiency of liver-derived endocrine IGF-I might delay aging, at least in terms of collagen aging.

Mean life span was 22.5 months in the control mice, which is approximately similar to the controls in *Igf1r* ^+/−^ mice studied by Holzenberger *et al.* [8], but lower than that in wildtype C57Bl/6J mice reported by the Jackson Laboratory (http://www.jax.org/). The shorter life span in our control mice compared to wildtype C57Bl/6J mice could be due to the genetic construction that the control mice were carrying (exon 4 of the IGF-I gene flanked by loxP sites) or due to differences in the environment or handling of the mice. In the present study, necropsies were not performed. However, in this as well as in our previous studies of LI-IGF-I^-/-^ mice of various ages, we have not observed any signs of a specific stress or disease in these mice [24], [27]–[30], [32]–[35].

In the previous study by Holzenberger *et al.*, low IGF-I activity in *Igf1r* ^+/−^ mice resulted in increased mean life span whereas the effect on maximal life span was moderate [8]. In LI-IGF-I^-/-^ mice, maximal lifespan was unaffected in the total group of mice and in male mice and only non-significantly tended to be increased in female mice. The possibility cannot be fully excluded that the effect on maximal life span was small because deficiency of liver-derived IGF-I only had a marginal effect on biological aging in late life, and that increased mean life span in LI-IGF-I^-/-^ mice mainly was due to increased resistance to stresses in their environment during their entire life span.

In conclusion, adult inactivation of liver-derived, endocrine IGF-I results in a modest but significant increase in mean life span, an effect that is more predominant in female than male LI-IGF-I^-/-^ mice. A contributing mechanism for the increased mean life span might be reduced body weight due to reduced body fat, which might partly result from increased energy expenditure during exercise. A consistent regulation of three genes in several tissues might directly or indirectly be of importance for the increased longevity in LI-IGF-I^-/-^ mice by modulating stress response and collagen aging.

## Materials and Methods

### Animals and serum IGF-I

The LI-IGF-I^-/-^ mice were generated as described previously (C57BL/6 background) [24], [27]–[30]. Mice homozygous for LoxP [58] and heterozygous for Mx-Cre [59] received three ip injections of polyinosinic-polycytidylic acid (PiPc; 6.25 µg/g body weight; Sigma-Aldrich Corp., Stockholm, Sweden) to induce expression of the Cre protein, thereby inactivating the IGF-I gene in the liver [59]. This treatment was given at one month of age to study life span, fertility, oxygen consumption at submaximal exercise, and gene expression, or at 12 months of age to measure food intake, oxygen consumption at rest, activity level, and body composition. PiPc-treated littermates, homozygous for LoxP but lacking Mx-Cre, were used as controls. Seven days after the PiPc injections, serum was obtained and assayed for IGF-I by a double-antibody IGF-binding protein-blocked RIA (Mediagnost, Tübingen, Germany). The animals had free access to fresh water and food pellets (B&K Universal AB, Sollentuna, Sweden). The ethical committee at the University of Gothenburg approved this study. Ethical approval number: 92-2004.

### Study design

Body weight and life span were determined in 137 (67 female and 70 male) control mice and 84 (31 female and 53 male) LI-IGF-I^-/-^ mice. Fertility was determined in 5- and 8-month-old female and male control and LI-IGF-I^-/-^ mice (n = 12–14 in each group). Energy expenditure was assessed in 24-month-old female control (n = 8) and LI-IGF-I^-/-^ (n = 5) mice and during submaximal exercise in 18-month-old female control (n = 7) and LI-IGF-I^-/-^ (n = 7) mice. Microarray analyses of gene expression in liver, skeletal muscle, heart, fat, and bone (vertebrae) were performed in female control (n = 6) and LI-IGF-I^-/-^ (n = 7) mice.

In additional experiments, liver-derived IGF-I was inactivated at 12 months of age. This late inactivation of IGF-I in hepatocytes resemble the age-related decline in serum IGF-I observed in elderly humans more closely than inactivation of liver-derived IGF-I in early adult life. Food intake, indirect calorimetry at rest, and spontaneous activity level were assessed in 13-month-old female control (n = 8) and LI-IGF-I^-/-^ (n = 8) mice one month following inactivation of liver-derived IGF-I and before there was any between-group difference in body weight. Dual-energy X-ray absorptiometry (DEXA) determined body composition in male control (n = 8) and LI-IGF-I^-/-^ (n = 6) mice at 18 months of age, 6 months following inactivation of liver-derived IGF-I at 12 months of age, and weights of fat pads were also determined.

### Fertility, body composition and food consumption

Fertility was assessed by mating female or male LI-IGF-I mice with control mice of opposite gender and the proportion of pregnant female mice and the litter size were determined.

Body composition was assessed using dual-energy X-ray absorptiometry (DEXA; PIXImus, Lunar Corporation, Madison, Michigan, USA). Total body fat and total body lean mass were measured from the lower level of the head to the inferior level of the first tail vertebra (the maximal scan area of the DEXA). Food intake and feces output were measured every 24 hours while mice were kept individually in metabolic cages for 5 days with free access to chow and drinking water, and the 3 last days were used for analyses.

### Activity measurement and indirect calorimetry

Mice were housed in separate chambers during these experiments. Spontaneous physical activity was measured using a photocell-based activity monitor (Opto-Max; Columbus Instruments, Columbus, OH). Interruption of four infrared photocells on the short side and eight photocells on the long side of the cage recorded the activity. Broken beams were recorded every 30 seconds for 2 hours, and the last hour was used for analysis.

The volume of O_2_ consumed (VO_2_) and CO_2_ produced (VCO_2_) were measured by an Oxymax system (Columbus Instruments) for 120 min at 30°C (thermoneutrality). A steady-state was reached after 90 min and the following 30 min between 90 and 120 min were used for analysis. VCO_2_ and VO_2_ were measured every sixth min as the mean of every second measurement. Respiratory exchange ratio (RER) was measured as the ratio of VCO_2_ to VO_2_.

### Energy expenditure during submaximal exercise

Mice were exercised on a motorized treadmill (Columbus Instruments) with an adjustable belt speed (0–100 m/min) and inclination (−10° to 25°). The treadmill was connected to the Oxymax system (Columbus Instruments) for measurement of energy expenditure by indirect calorimetry. The mice were encouraged to run by gentle tapping on their back. Before the experiments, all mice were acclimatized to the treadmill during a 3-day period with 5 min of rest and 5 min of running at 10 m/min and 0° inclination each day. On the test day, the mice rested for 1–2 h in the treadmill at room temperature (23°C) and then ran at a fixed speed of 10 m/min with an inclination of 0° for 30 min, followed by a 20 min post-exercise rest period.

### DNA microarray analysis and bioinformatics

RNA from liver, skeletal muscle, heart, fat (retroperitoneal fat) and bone (vertebrae) was extracted from 6 control and 7 LI-IGF-I^-/-^ mice by Tri Reagent (Sigma, St. Louis, MO, USA) and purified using spin columns from Rneasy Total RNA Isolation Kit (Qiagen, Chatsworth, CA, USA). For microarray analysis, the RNA samples were pooled three or two, resulting in three pools per group while for the confirmatory RT-PCR analyses individual mice (n = 6–7) were analyzed. The pooled RNA was reverse transcribed into cDNA, labeled, and analyzed by DNA microarray (MG-U74Av2 Array; Affymetrix, Santa Clara, CA, USA). The microarray data is MIAME compliant and can be accessed at EMBL-EBI ArrayExpress repository, ArrayExpress accession: E-MEXP-3026.

Scanned output files were analyzed using Affymetrix Micro Array Suite version 4.0.1 software (Affymetrix). To allow comparison of gene expression, the gene chips were globally scaled to an average intensity of 500. Each of the three LI-IGF-I^-/-^ chips was compared with the three control chips, generating a total of nine comparison files. Only the genes that were regarded as “changed” according to the Affymetrix algorithm in four to nine of the comparisons were selected for further analysis. We then calculated an average-fold change for the nine comparisons of the selected genes. For a gene to be regarded as consistently regulated in the LI-IGF-I^-/-^ mice, the average-fold increase or decrease of the nine comparisons was set as at least 1.5 fold in at least 4 of the 5 analyzed organs.

### Real-time PCR analysis

To confirm the microarray findings, we performed RT-PCR analyses on skeletal muscle (triceps) samples from individual mice (n = 6–7) (ABI Prism 7700 Sequence Detection System (PE Applied Biosystems, Stockholm, Sweden). We used FAM-labeled probes specific for heat shock protein 1A (Mm01159846_s1) and 1B (Mm03038954_s1), connective tissue growth factor (Mm01192931_g1), CCAAT/enhancer protein delta (Mm00514291_s1), and IGF-I receptor (Mm00802831_m1). The reporter fluorescent dye VIC, specific for 18S rRNA, was included as an internal standard. The cDNA was amplified at 1 cycle at 50 C for 2 min and 95 C for 10 min, followed by 40 cycles at 95 C for 15 sec and 60 C for 1 min. We calculated the mRNA amount of each gene using the standard curve method (multiplex reaction, User Bulletin no. 2, PE Applied Biosystems).

### Statistical analyses

All descriptive statistical results are presented as the mean ± SEM. Between-group differences were calculated using unpaired *t*-tests. Differences between survival curves were determined using Cox's test. Maximum lifespan was calculated as the number of mice that were alive (and number dead) at the age at which 90% of the joint distribution (LI-IGF-I^-/-^ plus control mice) had died [60], [61]. A two-tailed p<0.05 was considered significant.
